# SHBG141–161 Domain-Peptide Stimulates GPRC6A-Mediated Response in Leydig and β-Langerhans cell lines

**DOI:** 10.1038/s41598-019-55941-x

**Published:** 2019-12-19

**Authors:** Luca De Toni, Diego Guidolin, Vincenzo De Filippis, Daniele Peterle, Maria Santa Rocca, Andrea Di Nisio, Maurizio De Rocco Ponce, Carlo Foresta

**Affiliations:** 1 University of Padova, Department of Medicine and Unit of Andrology and Reproductive Medicine, 35128 Padova, Italy; 2 Unversity of Padova, Department of Neuroscience and Section of Anatomy, 35128 Padova, Italy; 3 University of Padova, Department of Pharmaceutical and Pharmacological Sciences, 35131 Padova, Italy; 4 Familial Cancer Clinic, Veneto Institute of Oncology (IOV-IRCCS), 35128 Padova, Italy

**Keywords:** Computational models, Metabolic syndrome

## Abstract

GPRC6A is acknowledged as a major regulator of energy metabolism and male fertility through the action of undercarboxylated osteocalcin (ucOCN), representing a possible therapeutic target. We recently showed that the sex hormone-binding globulin (SHBG) binds to GPRC6A through the likely involvement of the 141–161 domain. To confirm this model, here we investigated the possible binding and agonist activity of SHBG(141–161) domain-peptide (SHBG_141–161_) on GPRC6A. The binding of SHBG_141–161_ to GPRC6A and downstream dissociation from G_αi_(GDP) protein was computationally modelled. SHBG_141–161_ was obtained by solid-phase synthesis, characterized by circular dichroism (CD) and the receptor binding was assessed by displacement of ucOCN on HEK-293 cells transfected with *GPRC6A* gene. Agonist activity of SHBG_141–161_ was assessed on Leydig MA-10 and Langerhans β-TC6 cell lines through the GPRC6A-mediated release of testosterone (T) and insulin. SHBG_141–161_ was predicted to bind to GPRC6A and to reduce the affinity for G_αi_(GDP) at computational level. Conformational properties and binding to GPRC6A of the synthetic SHBG_141–161_ were confirmed by CD and displacement experiments. SHBG_141–161_ stimulated cell secretion of T and insulin, with dose dependency from 10^−13^ to 10^−11^M for T release (respectively P = 0,041 10^−13^M; P = 0,032 10^−12^M; P = 0,008 10^−11^M *vs* basal) and for 10^−12^ to 10^−10^M for insulin (respectively P = 0,041 10^−12^M; P = 0,007 10^−11^M; P = 0,047 10^−10^M; P = 0,045 *vs* basal). Blockade with anti GPRC6A IgG abolished the response to SHBG_141-161_, suggesting agonist specificity. SHBG_141–161_ showed stimulating activity on GPRC6A, representing a template peptide with possible therapeutic use for metabolic and endocrine disorders.

## Introduction

Recent experimental and clinical evidence support the existence of a novel endocrine axis connecting the bone to other metabolism-relevant organs, such as testis, pancreas, muscle and liver^1^. According to this endocrine pathway, the osteoblast-derived protein osteocalcin (OCN) is released into the bone extracellular matrix in its full γ-carboxylated form (cxOCN), participating of the deposition of hydroxyapatite. Insulin-mediated bone remodeling, through acidification of resorption lacunae, allows desorption of undercarboxylated-OCN (ucOCN) from calcified tissue and access of the protein into the bloodstream where it activates target tissues expressing GPRC6A, the established molecular target of ucOCN^2^. GPRC6A is a G-protein coupled receptor with wide metabolic implication, among which the stimulation of insulin secretion from β-cells and the production and release of testosterone (T) from Leydig-cells. Accordingly, animal models knock-out for *Ocn* or *Gprc6a* genes are phenocopies, showing glucose intolerance, impaired insulin sensitivity, increased adiposity and hypogonadism-related infertility in males^3^. Importantly, this pattern was confirmed in humans, supporting the conservation of this pathways also in mammals of higher order^4^.

As a metabolite sensing receptor, one of the peculiarity of GPRC6A is that of being activated by a number of ligands in addition to ucOCN, such as metal cations, like calcium, zinc, and magnesium, basic amino acids, like L arginine, L-lysine, and L-ornithine^5^. Of note, GPRC6A has also been recognized as the mediator of non-genomic action of T^6^, suggesting the pharmacological opportunity to regulate androgen activity in a non-steroidal manner^7,8^. To this regard, our group recently reported that sex hormone-binding protein (SHBG), the major blood carrier of T, is able to bind the extracellular domain of GPRC6A on the putative binding site of ucOCN thanks to common structural moieties of the two proteins^9^. In particular, the predicted interface exploited by SHBG to bind the extracellular domain of GPRC6A corresponded to the sequence Leu146- Pro159 in the loop region Ile141-Leu161, connecting β-strands 11 and 12 on the opposite side with respect to the steroid-binding site in SHBG^9^. In order to validate this molecular model, in this study we characterized the functional properties of the synthetic SHBG(141–161) peptide (SHBG_141–161_) on GPRC6A. To this aim, we firstly investigated the possible binding and agonist activity of SHBG_141–161_ on the extracellular domain of GPRC6A through a computational approach. Subsequently, we verified experimentally both the possible binding activity of SHBG_141–161_ on GPRC6A and its role as agonist on Leydig and β-Langerhans cell lines as target cell populations constitutively expressing GPRC6A.

## Results

### Molecular modeling

**Figure 1:**
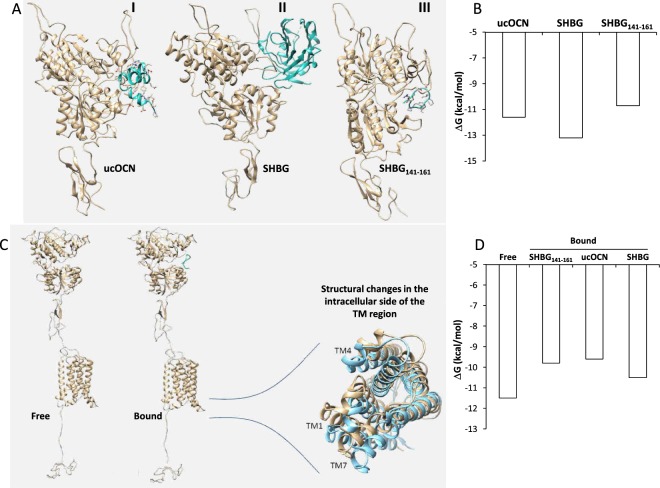
(**A)** Lowest energy docked structures between extracellular domain of GPRC6A and respectively undercarboxylated osteocalcin (ucOCN, panel I), sex hormone binding globulin (SHBG, where only the interacting subunit of the dimer is shown, panel II) and SHBG(141–161)-domain peptide (SHBG_141–161_, panel III). (**B**) Estimated changes in free energy (ΔG) upon binding of GPRC6A with the aforementioned ligands ucOCN, SHBG and SHBG_141–161_. (**C**) Estimated conformation of the intracellular side of the transmembrane (TM) region of GPRC6A in its free form (Free, amber coiled structure in the inset) and upon the binding with SHBG_141–161_ (Bound, cyan coiled structure in the inset). (**D**) Estimated ΔG of the binding of GPRC6A with the G_αi_(GDP) protein in its Free form and in complex with either ucOCN, SHBG and SHBG_141–161_.

The best solutions of the modeling procedure suggested for the SHBG_141–161_ peptide a free-coil configuration as the most probable, similar to the configuration it assumes when inserted in the SHBG molecule (Fig. 1A).

Docking simulations (Fig. 1A) indicated that the three considered ligands (SHBG_141–161_, SHBG, ucOCN) bind human GPRC6A receptor model almost in the same region (involving Phe281-Asn294, Lys310-Pro315, His360-Tyr366, Arg399-Phe402) of the extracellular Venus flytrap with significant affinity as indicated by their Gibbs free energy changes upon binding (Fig. 1B). Of note, the model of binding between SHBG_141–161_ and the receptor involved a number of noncovalent interactions, including hydrogen bonding and hydrophobic interactions, detailed in respectively Supplemental Figures S2 and S3.

As a consequence of complex formation, conformational changes in the transmembrane domain of the receptor were also predicted (Fig. 1C), suggesting they could modify the affinity of the receptor for the G_αi_(GDP) protein and influence the dynamics of signal transduction. Interestingly, G_αi_(GDP) protein affinity for the receptor liganded by ucOCN or SHBG_141–161_ was predicted to be lower than the affinity for the unbound receptor as expressed by the free energies of the complex (Fig. 1D), a finding consistent with an increased probability of signal transduction from GPRC6A. This change in G_αi_(GDP) protein affinity resulted less evident when GPRC6A liganded to SHBG was considered.

### Chemical synthesis, conformational properties and binding activity of SHBG_141–161_ peptide

**Figure 2:**
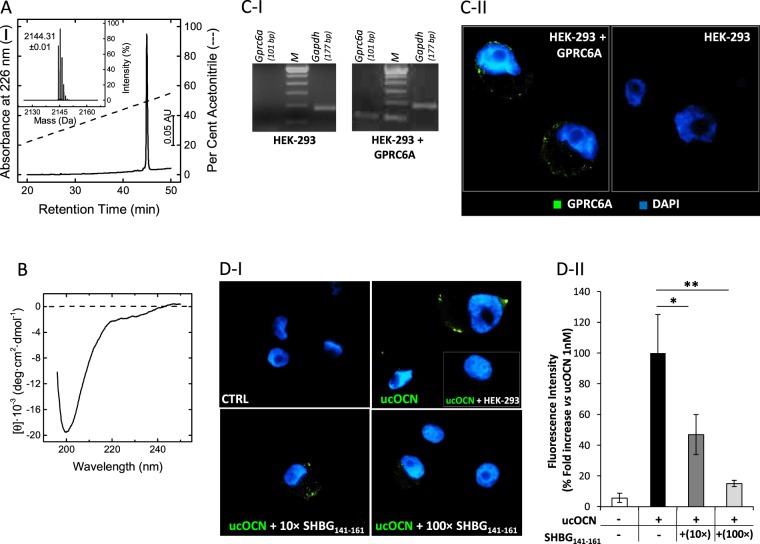
Chemical and conformational characterization of SHBG(141–161) domain-peptide (SHBG_141–161_). (**A**) Purity check of the synthetic SHBG(141–161). An aliquot (50 μl, 25 μg) of the purified peptide was analysed by RP-HPLC on Grace-Vydac C18 (4.6 × 250 mm, 5 μm) eluted with a linear acetonitrile–0.1% TFA gradient from 5 to 55% in 45 min at a flow rate of 0.8 ml/min. The peptide material eluted in correspondence of the major chromatographic peak was collected and analysed by high-resolution MS (Inset) yielding a monoisotopic mass value of 2154.31 a.m.u. (**B**) Far-UV circular dichroism spectra of SHBG_141–161_. The spectrum was recorded at 25 °C in PBS (20 mM sodium phosphate, pH 7.4, 0.1 M NaCl) at a peptide concentration of 70 μg/ml. Binding activity of SHBG_141–161_ on GPRC6A. (**C**) Transfection of human-*Gprc6a* gene in HEK-293 cells. (**C**–**I**) Analysis of real-time PCR products of *Gprc6a* gene in control cells with empty vector (HEK-293) and HEK-293 cells transfected with *Gprc6a* gene (HEK-293 + GPRC6A) and. Human *Gapdh* gene served as housekeeping. Laddering marker (M) was used for verify the molecular weight of amplification products. (**C**–**II**) Representative immunofluorescence staining for GPRC6A (green) in HEK-293 + GPRC6A and controls HEK-293. Cells were counterstained with DAPI (blue). (**D)** Displacement experiments of undercarboxylated osteocalcin (ucOCN) with SHBG_141–161_. (**D**–**I**) Representative staining of 1 nM labelled-ucOCN (green) on HEK-293 + GPRC6A, co-incubated with SHBG_141–161_ at the concentration of 0 nM (CTRL), 10 nM (10×) and 100 nM (100×). Image in the inset reports the staining of 1 nM labelled-ucOCN on HEK-293. (**D**–**II**) Image analysis for labelled-ucOCN staining intensity in the aforementioned conditions. Data are reported as fold increase compared to 1 nM labelled-ucOCN condition and represent mean values of 3 independent experiments. Significance: *P < 0,05 and **P < 0,01 among indicated conditions.

The peptide encompassing the amino acid sequence 141–161 (IALGGLLFPASNLRLPLVPAL) and corresponding to the loop region connecting β-strands 11 and 12 in the SHBG crystallographic structure [1D2S.pdb;^10^] was produced by solid-phase chemical synthesis using the Fmoc-chemistry. The peptide was purified to homogeneity (>98%) by semi-preparative reversed-phase high performance liquid chromatography (RP-HPLC) and its chemical identity was established by high-resolution mass spectrometry (MS), yielding a monoisotopic mass value of 2144.38 a.m.u which is identical to the theoretical value deduced from the amino acid sequence of the peptide, 2144.31 a.m.u. (Fig. 2A). The conformational properties of the synthetic peptide were investigated by far-UV circular dichroism (Fig. 2B), displaying a single minimum centered at 200 nm, typical of a polypeptide chain in a random or non-regular conformation^11^, similar to the structure that the sequence 141–161 assumes in the 3D structure of the parent SHBG. With respect to this point, the concomitant presence of two consecutive Glycine residues and three Prolines in SHBG_141–161_ sequence impair acquisition of ordered α-helical or β-sheet secondary structure^12,13^.

The binding of SHBG_141–161_ to GPRC6A was assessed by displacement experiments as previously described^3^. To this aim, HEK-293 cells were transfected with human *Gprc6a* gene (Fig. 2C-I,II). The efficiency of the procedure was evaluated by real-time PCR, allowing the identification of a specific signal for *Gprc6a* gene only in transfected cells. Accordingly, a specific membrane signal was observed by immunostaining only in transfected cells. On these bases, HEK-293 cells transfected with *Gprc6a* gene were incubated with 1 nM labelled ucOCN and SHBG_141–161_ at the concentration of 0, 10 nM (10×) and 100 nM (100×) (Fig. 2D-I,II). Incubation with labelled ucOCN alone resulted in a clear membrane signal associated with GPRC6A expression, as suggested by the absence of significant staining in non-transfected cells (inset in ucOCN image). Co-incubation with SHBG_141–161_ at increasing concentration resulted in a progressive reduction of the signal for labelled ucOCN. Image analysis for fluorescence intensity showed a significant reduction of the staining signal for ucOCN when co-incubated with 10× (P = 0,021) or 100× (P = 0,006) SHBG_141–161_, compared to the presence of ucOCN alone.

Taken together, these results indicate that SHBG_141–161_ compete with ucOCN for the binding to GPRC6A and suggest a role for the binding of the synthetic peptide to the receptor.

### SHBG_141–161_ stimulates secretory function in GPRC6A expressing cells

**Figure 3:**
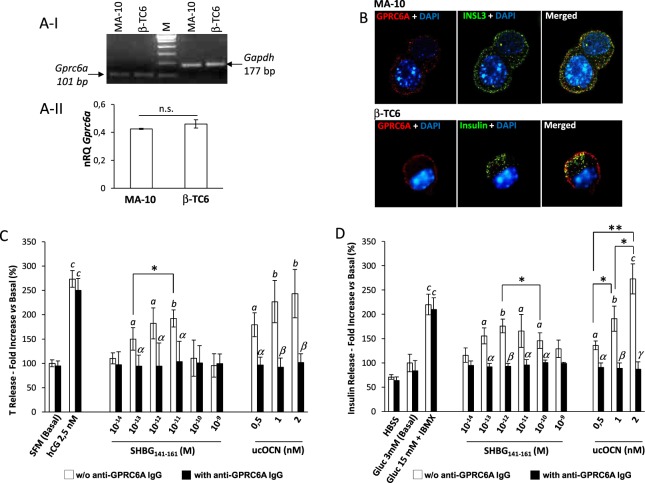
Stimulating activity of SHBG(141–161)-domain peptide (SHBG_141–161_) on GPRC6A-expressing cells. (**A**–**I**) Analysis of real-time PCR products of *Gprc6a* gene in Leydig MA-10 (MA-10) and Langerhans β-TC6 (β-TC6) cells. Human-*Gapdh* served as housekeeping. Laddering marker (M) was used for verify the molecular weight of amplification products. (**A**–**II**) Quantitative comparison of Gprc6a gene expression between the two cell lines. Expression data are normalized on *Gapdh* gene. Significance: n.s. = P > 0,05. (**B**) Representative immunostaining for phenotype analysis of MA-10 and β-TC6. Panel MA-10) Double immunostaining for GPRC6A (red) and insulin-like peptide 3 (INSL3, green). Panel β-TC6) Double immunostaining for GPRC6A (red) insulin (green). Cells were counterstained with DAPI (blue). Images are representative of 3 independent experiments. (**C**) Quantification of testosterone (T) release after stumulation of MA-10 cells with human-chorionic gonadotropin (hCG), SHBG_141–161_, and undercarboxylated osteocalcin (ucOCN) at the indicated concentrations. Cells were stimulated in either absence (white bars) or presence (black bars) of blocking anti human GPRC6A polyclonal antibodies (anti-GPRC6A IgG). Data are reported as fold increase compared to serum free-medium as basal condition [SFM (Basal)]. (**D**) Quantification of insulin release after stumulation of β-TC6 cells with 15 mM glucose + 100 μM 3-Isobutyl-1-methylxanthine (Gluc 15 mM + IBMX), SHBG_141–161_ and ucOCN at the indicated concentrations. Cells were stimulated in either absence (white bars) or presence (black bars) of blocking anti-GPRC6A IgG. Data are reported as fold increase compared to 3 mM glucose HBSS medium as basal condition [Gluc 3 mM (Basal)]. Results are representative of five independent experiments. Significance *a* = P < 0,05, *b* < 0,01 and *c* < 0,001 *vs b*asal condition, respe*c*tively. *α* = P < 0,05 and β = P < 0,01 *vs* corresponding condition in absence of anti GPRC6A IgG, respectively. *P < 0,05 and **P < 0,01 among indicated conditions.

In order to evaluate whether, in addition to the binding activity, SHBG_141–161_ might act as a GPRC6A agonist, we investigated the effect of GPRC6A stimulation on target cell populations with constitutive expression of this receptor. Accordingly, we focused on MA-10 and β-TC6 cell lines, which are known to express GPRC6A that mediates the production and secretion of testosterone and insulin, respectively, and to be responsive to human ucOCN^14–17^. Since prolonged culture conditions may alter the molecular phenotype of cells^18^, the consistent expression of GPRC6A was firstly assessed. Gene expression analysis showed that both MA-10 and β-TC6 had comparable levels of *Gprc6a* transcripts (Fig. 3A-I,II). Furthermore, immunofluorescence was performed in order to assess both the phenotypic identity of cells and the consistent expression of GPRC6A. MA-10 cells were double stained for GPRC6A and insulin-like peptide 3 (INSL3), a peptide hormone exclusively produced by Leydig cells^19^. Accordingly, signals for GPRC6A showed an essentially membrane localization whilst INSL3, being both a constitutive and secretory protein, showed a mixed cytoplasm/membrane staining (Fig. 3B-panel MA-10). On the other hand, phenotypic staining for insulin in β-TC6 showed an expected dotted signal on cell cytoplasm coupled with a clear membrane staining for GPRC6A (Fig. 3B-panel β-TC6).

On these bases, both MA-10 and β-TC6 were stimulated with SHBG_141–161_ (10^−14^–10^−9^ M), ucOCN (0,5 −2 nM). In addition, 2,5 nM human chorionic gonadotropin (hCG) and 100 μM 3-isobutyl-1-methylxanthine (IBMX) in 15 mM glucose were used as reference agonists to induce maximal release of T and insulin, respectively, as previously described^20,21^.

In MA-10 cells (Fig. 3C), hCG efficiently stimulated T release compared to basal-unstimulated conditions (P < 0,001), confirming the functional efficiency of the cell line. Incubation with SHBG_141–161_ was associated with an increase of T release, particularly at concentration from 10^−13^ to 10^−11^M that showed significant difference compared to basal (respectively; P = 0,041 10^−13^M; P = 0,032 10^−12^M; P = 0,008 10^−11^M *vs* basal). In addition, T release at 10^−11^M was higher than at 10^−13^M (P = 0,045) suggesting a dose-effect dependency on SHBG_141–161_ concentration. On the other hand, incubation with ucOCN was associated with a progressive and significant increase of T release compared to basal condition (respectively P = 0,015 0,5 nM; P = 0,009 1 nM; P = 0,006 2 nM *vs* basal).

Also in β-TC6 cell line (Fig. 3D), whose full function was confirmed by strong insulin secretion induced by IBMX/glucose compared to basal conditions (P < 0,001), a similar trend was observed since SHBG_141–161_ increased insulin secretion. In particular, incubation with SHBG_141–161_ at concentration ranging from 10^−13^ to 10^−10^ M was associated with significant increased insulin levels in culture supernatants compared to basal (respectively: P = 0,041 10^−13^M; P = 0,007 10^−12^M; P = 0,047 10^−11^M; P = 0,045 *vs* basal). In addition, insulin release at 10^−12^M was higher than at 10^−10^M (P = 0,039) suggesting a dose-effect dependency on SHBG_141–161_ concentration. On the other hand, incubation with ucOCN was associated with a significant and dose dependent increase of insulin release compared to basal condition (respectively: P = 0,011 0,5 nM; P = 0,008 1 nM; P < 0,001 2 nM *vs* basal; P = 0,042 0,5 nM *vs* 1 nM; P = 0,028 1 nM *vs* 2 nM; P = 0,006 0,0 nM *vs* 2 nM).

Preliminary experiments on both cell lines showed that concentrations of SHBG_141–161_ greater than 10^−9^ M were not effective (data not shown), suggesting non-linear response to SHBG_141–161_. Importantly, receptor blockade experiments by pre-incubation with anti-GPRC6A polyclonal antibodies, completely abolished the agonist activity of both ucOCN and SHBG_141–161_ (Fig. 3C,D).

**Figure 4:**
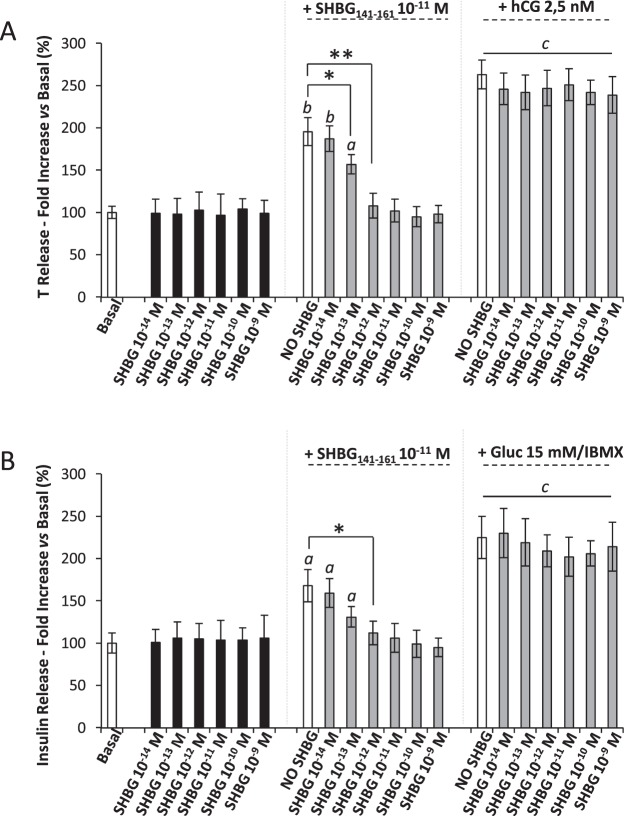
Stimulating activity of steroid hormone binding globulin (SHBG) on GPRC6A-expressing cells. Leydig MA-10 **(A**) and Langerhans β-TC6 (**B**) cell lines were incubated as described in the method section with SHBG, at the indicated concentrations, and culture supernatant was evaluated for, respectively, testosterone (T) or insulin levels. In pre-incubation experiments, both cell types were pre-incubated for 1 hour at 37 °C with SHBG at the indicated concentration and then stimulated with SHBG(141–161)-domain peptide (SHBG_141–161_ 10^−11^M) at the concentration of 10^−11^M. T and insulin levels were then quantified. Human-chorionic gonadotropin 2,5 nM (hCG 2,5 nM) and 15 mM glucose + 100 μM 3-Isobutyl-1-methylxanthine (Gluc 15 mM/IBMX) were also used as reference stimulation for respectively MA-10 and β-TC6 after pre-incubation with SHBG at the indicated concentrations. Results are representative of five independent experiments Significance *a* = P < 0,05, *b* < 0,01 and *c* < 0,001 *vs* basal condition, respectively. *P < 0,05 and **P < 0,01 among indicated conditions.

Finally, the possible agonist activity of the parent protein SHBG was assessed by stimulating MA-10 and β-TC6 cells at the same range of concentrations of 10^−14^-10^−9^M used for SHBG_141–161_ (Fig. 4A,B). SHBG was ineffective in inducing either T or insulin release at any of the concentration tested in both cell lines. However when either cell lines, stimulated with SHBG_141–161_ 10^−11^M, were pre-incubated with increasing concentration of SHBG for 1 hour at 37 °C, a progressive reduction of T and insulin release was observed. In particular, pre-incubation with SHBG 10^−12^M was associated with a complete blunt of the secretory stimulating activity of SHBG_141–161_ in both MA-10 and β-TC6 cells. In MA-10 cells a significant reduction of T release, associated with incubation with SHBG_141–161_, was also observed after pre-incubation with SHBG even at the concentration of 10^−13^M (P = 0,038). In addition SHBG showed negligible effect on T and insulin release, induced by specific stimulatory agents on MA-10 and β-TC6 cells (respectively hCG and IBMX/glucose), at any of the concentration tested.

Taken together, these data suggest that SHBG_141–161_ stimulates GPRC6A-mediated response in cell lines constitutively expressing the receptor. Pre-incubation experiments with parent SHBG support the competition with SHBG_141–161_ on the same target.

## Discussion

In this study we provide evidence that the synthetic peptide encompassing the amino acid sequence 141–161 of human SHBG owns ligand capacity on GPRC6A. Furthermore, stimulation of cell populations stably expressing GPRC6A with SHBG_141–161_, associates with the activation of GPRC6A-mediated cell function such as T and insulin release from Leydig and Langerhans β-cells cell lines, respectively. To the best of our knowledge, this represents the first study reporting the agonistic activity of a peptide of human origin on GPRC6A.

Since its characterization in 2005, GPRC6A gained growing interest because of its deep involvement in energy metabolism and endocrine regulation of male fertility, particularly in animal models [reviewed in^1^]. Despite some controversies are still to be addressed, a parallel regulating role of GPRC6A has been claimed in humans^22^. On these bases, GPRC6A attracted attention as a molecular target to pursue therapeutic strategies for metabolic and endocrine disorders. To this regard, through a target-based computational approach, Pi *et al*. identified novel low molecular weight-compounds showing agonist activity for GPRC6A^7^. Interestingly, a candidate tri-phenol compound showed stimulatory effect on insulin secretion both *in vitro* and *in vivo* on animal models. However, no modulatory effect on T release was investigated^7^.

If on one hand these represent encouraging results about the reliability of GPRC6A as therapeutic target, on the other hand, drug peptides and biologics drown great interest for their favorable safety profile, target specificity and pharmacokinetics compared with traditional small-molecule drugs^23^. To this regard, the metabotropic receptor GPRC6A has been widely investigated as the mediator of non-genomic effects of androgens^6^. Since androgens have been shown to trigger specific signaling pathways through the binding to SHBG and the membrane-SHBG receptor^24–26^, we recently suggested that SHBG may act as a molecular primer, binding to the orthosteric site of the extracellular domain of GPRC6A and activating the downstream signaling of the receptor through the subsequent interaction with T^9^. Consistent with this model that views a role of SHBG in human metabolism, it should be noted that low circulating levels of SHBG have been identified as a strong risk factor for type 2 diabetes in both women and men, with possible direct regulation of glucose transporters expression in peripheral tissues^27,28^. Importantly, computational analysis of SHBG and ucOCN, the major endogenous agonist of GPRC6A, showed similar structural moieties of the two proteins. In particular, the interface exploited by SHBG to bind the extracellular domain of GPRC6A and corresponding to the loop domain Ile141-Leu161, exhibited a high similarity score with OCN^9^. Consequently, the SHBG(141–161)-domain peptide was expected to maintain the binding ability to GPRC6A. In the present study, this hypothesis was assessed through a combined computational and experimental approach. The possible docking of SHBG_141–161_ to GPRC6A was firstly modelled and compared to that of both the parent molecule, i.e. SHBG, and the endogenous agonist, i.e. ucOCN. To this regard, the accurate prediction of a peptide conformation may represent a limiting step, influencing in turn charge distribution and protein-protein surface interaction^29^. Computer-based prediction failed to identify a true secondary structure for SHBG_141–161_, attributing a conformation largely random or unstructured, similar to that displayed by the same domain within the whole SHBG^10^. This structural hypothesis was experimentally confirmed by CD data on the synthetic peptide, validating the consistency on the computational modelling. Interestingly, SHBG_141–161_ was predicted to bind the extracellular domain of GPRC6A with an affinity similar to that of ucOCN, but lower than SHBG. This evidence suggested major differences among the three ligands as agonists, as recently proposed^30^. Accordingly, the dissociation model from G_αi_(GDP) showed a remarkable reduction of the G-protein/GPRC6A complex stability after the binding with either ucOCN or SHBG_141–161_, but not with SHBG. The suggested role of SHBG_141–161_ as agonist on GPRC6A was then experimentally verified through the GPRC6A-mediated T and insulin release from respectively MA-10 and β-TC6 cells, an effect completely abolished by the blockade model with anti-GPRC6A polyclonal antibodies. Notably, SHBG_141–161_ showed a non-linear dose-response effect on either cell lines. This evidence is not uncommon among G-protein coupled receptors (GPCRs), for which biphasic, monophasic or bell-shaped dose-response curves have been described^31,32^. This behavior has been recently modelled as the result of the formation of oligomers, affecting the binding of extracellular ligands to GPCRs and enabling the ligands to produce a wider and more complex range of functional responses (reviewed in^33^). Accordingly, dimerization of GPRC6A has been hypothesized since early findings and such functional organization might explain the apparently anomalous response to SHBG_141–161_ peptide^30,34^. In addition, the model viewing SHBG and SHBG_141–161_ converge on the same binding site of GPRC6A through shared structural moieties, was supported by pre-incubation experiments with parent SHBG. In particular, although SHBG_141–161_ effectively competes with ucOCN in GPCR6A binding, it does not completely abrogate ucOCN interaction. Hence, from this standpoint, we can conclude that SHBG_141–161_ displays partial competition activity. On the other hand, SHBG showed non-agonist activity on GPRC6A-expressing cells but was able to reduce cell response to SHBG_141–161_ in a dose dependent manner, an evidence compatible with the simple binding observed through immunoprecipitation assays in our previous study^9^. In particular in MA-10 cells, stimulating effects of SHBG_141–161_ 10^−11^M were significantly blunted by SHBG at the concentration of 10^−13^M, supporting a nearly 100 fold difference in affinity as extrapolated from the computational data of Gibbs free energy changes.

We acknowledge the essentially pre-clinical value as the major drawback of this study. In addition, we did not evaluate the downstream signaling of GPRC6A subtending functional assays on either Leydig or Langerhans β-cell lines. However, there is substantial literature supporting a major role of respectively adenylate cyclase/cAMP pathway in GPRC6A-mediated release of T and of phospholipase C/DAG pathway in GPRC6A-mediated release of insulin^3,15^. Furthermore, our data suggest that SHBG_141–161_ represents an interesting template peptide owning both binding and stimulating activity on GPRC6A. Furthermore, we report the stimulatory effect on the steroidogenic activity of Leydig cells of the synthetic peptide. This represents an unprecedented issue that opens to possible novel scenarios for the treatment of male infertility based on endocrine derangements. To this regard, further investigation on the structure-activity relationship of SHBG_141–161_ are warranted to improve its agonist activity, as recently adopted for other peptides of potential therapeutic interest^35^.

In conclusion, computational docking analysis of SHBG(141–161)-domain peptide showed ligand and agonist activity of GPRC6A, an evidence confirmed experimentally by GPRC6A-mediated release of testosterone and insulin, respectively, from Leydig and Langherans β-Cell lines, stimulated with the synthetic peptide. This represents a possible novel pharmacologic approach for the treatment of metabolic and endocrine derangements through the activation of GPRC6A-regulating effects.

## Methods

### Molecular modeling

The applied modeling procedure involved three stages. At the first stage, since the crystal structure of GPRC6A was not reported yet, a 3D structure of the receptor was generated, based on receptor homology with known crystalized GPCRs^30^. After retrieving the FASTA sequence of the human GPRC6A (code: Q5T6XS) from the UniProtKB database (https://www.uniprot.org), the GalaxyWEB server (http://galaxy.seoklab.org) was used to obtain homology models of the receptor^36^ and the predicted structure exhibiting the highest score was considered for further processing. 3D structures of SHBG_141–161_ were obtained by using the Pep-Fold 2.0 software for de novo peptide structure prediction^37^ freely available at http://bioserv.rpbs.univ-paris-diderot.fr/services/PEP-FOLD. At the second step fully flexible docking/binding simulations were performed involving the GPRC6A model as receptor and the models of three ligands, namely the crystallographically assessed structure of SHBG (PDB code: 1D2S)^10^, and the predicted models for ucOCN^9,38^ and SHBG_141–161_. The online Galaxy 7TM specialized server (http://galaxy.seoklab.org) was used for docking and the solutions exhibiting the highest predicted affinity were structurally refined by using the GalaxyRefineComplex method^39^ to capture conformational changes upon binding. Finally, at the third step, G_α_ protein interaction with the intracellular domain of the refined liganded structures of GPRC6A was analyzed. The 3D structure of G_αi_(GDP) was retrieved from the RCSB Protein Data Bank (https://www.rcsb.org; PDB code: 6DDE) and docking with the ligand-receptor structures obtained from the above step was performed by using the Hex 8.0.0 software (http://hex.loria.fr/). The change in free energy upon binding was then estimated by using the PRODIGY web service (https://nestor.science.uu.nl/prodigy/)^40^.

### Chemicals

Human ucOCN was obtained from chemical decarboxylation of cxOCN, purchased from Bachem AG (Bachem, Bubendorf, Switzerland), as previously described^14^. For displacement experiments, an aliquot ucOCN underwent chemical biotinylation as previously described^9,41^. Unliganded human SHBG was purchased by Affiland (Liège, Belgium). Human chorionic gonadotropin Gonasi was purchased by IBSA Farmaceutici Srl (Italy).

### Chemical synthesis and characterization of SHBG[141–161)-domain peptide

The peptide sequence 141–161 of SHBG (IALGGLLFPASNLRLPLVPAL) was synthesized by the solid‐phase method using the 9‐fluorenylmethyloxycarbonyl (Fmoc) strategy on a model PS3 automated synthesizer from Protein Technologies International (Tucson, AZ)^42^. The peptide chain was assembled stepwise on a benzyloxybenzyl (Wang)-resin (Novabiochem, Switzerland) derivatized with Fmoc‐Leu (0,24 mequiv g^−1^); tert‐butyl side‐chain protecting group was used for Ser; triphenylmethyl for Asn and 2,2,4,6,7‐pentamethyldihydrobenzofuran‐5‐sulfonyl (Pbf) group was used for Arg. Removal of Nα‐Fmoc‐protecting groups was achieved by treatment for 20 min with 20% piperidine in N‐methylpirrolidone. Standard coupling reactions were performed with 2‐(1 H‐benzotriazol‐1‐yl)‐1,1,3,3‐tetramethyluronium hexafluorophosphate (HBTU) and 1 H‐hydroxy‐benzotriazole (HOBt) as activating agents, with a fourfold molar excess of Nα‐Fmoc‐protected amino acids (Novabiochem) in the presence of diisopropylethylamine. For double couplings at peptide bonds involving Val, Ile, Leu, and Phe, the stronger activator 2‐(7‐aza‐1 H‐benzotriazol‐1‐yl)‐1,1,3,3‐tetramethyluronium hexafluorophosphate was used (HATU). After peptide assembly was completed, the side chain‐protected peptidyl resin was treated for 120 min at room temperature with a mixture of TFA/H2O/ethandithiol/triisopropylsilane (90:5:4:1 v/v/v/v). The resin was removed by filtration, and the acidic solution, containing the unprotected peptide, was precipitated with ice‐cold diethylether and then lyophilized. The crude peptide was purified (>98%) by a semi-preparative RP‐HPLC on a Grace‐Vydac (Hesperia, CA) C‐18 column (1 cm × 25 cm, 5‐μm particle size) eluted with a linear acetonitrile–0.1% TFA gradient from 5 to 55% in 45 min at a flow rate of 1.5 ml/min and recording the absorbance of the effluent at 226 nm. The peptide material eluted in correspondence of the major chromatographic peak was collected, lyophilized, and analyzed by high-resolution mass spectrometry, using a Waters (Milford, MO, USA) Xevo-G2S Q-TOF mass spectrometer. For data acquisition and analysis, the software programs MassLynx 4.1 and BioPharmaLynx were used, respectively (Waters).

The conformational properties of SHBG_141–161_ were investigated by far-UV circular dichroism (CD) on a Jasco J-710 spectropolarimeter, equipped with a Peltier temperature control system. The molar concentration of the peptide was determined spectrophotometrically by recording the absorbace at 257 nm, using an absorptivity value for phenylalanine of 200 M-1·cm-1. CD spectra were recorded at 25 °C in a 1-mm pathlength quartz cell at a scan-speed of 10 nm/min, with a response time of 16 sec, and resulted from the average of two accumulations after baseline subtraction. CD signal was expressed as the mean residue ellipticity (θ) = θobs·MRW/(10·l·c), where θobs is the observed signal in degrees, MRW is the protein mean residue weight, l is the cuvette pathlength in cm, and c is the protein concentration in g/ml.

### Cell culture, reagents, and antibodies

Human embryonic kidney (HEK)-293T cells (passage number 11 to 15) were obtained from the American Type Culture Collection (ATCC®-LGC Standards Sesto San Giovanni, MI, Italy) and cultivated in DMEM [ATCC®; RRID 30–2002) and supplemented with 10% fetal bovine serum (Euroclone, Milano, Italy; RRID: ECS0180L) and 1%antibiotic/antimycotic mixture in a humidified incubator at 37 °C with 5% CO_2_. Expression vector containing wild-type (WT) human GPRC6A cDNA (OriGene Technologies, Inc) was used according to manufacturer’s instruction. Briefly, cultured HEK- 293 T cells at 70% of confluence were transfected by FuGENE 6 (Roche Diagnostics) with 1 μg/well of GPRC6A-pcDNA and maintained for 48 hours, followed by overnight starving in serum-free condition to achieve quiescence. HEK-293T cells transfected with empty vector were used as negative controls. Starved cells were fixed in 1% paraformaldehyde and used for binding assessment by incubation for 1 h at room temperature with biotinylated ucOCN, with or without SHBG_141–161_ in ice-cold-binding buffer (50 mM Tris-HCl [pH 7.4], 10 mM MgCl2, 0.1 mM EDTA, and 0.1% BSA) according to the different experimental conditions used. After extensive washing in cold PBS, samples were incubated with streptavidin-Fluorescein isothiocyanate (FITC) (dilution 1:200; Sigma-Aldrich; RRID: S3762). Evaluation of GPRC6A expression by immunofluorescence was performed as described for β-TC6 (see below). Computer assisted image analysis was performed by the ImageJ image analysis software, powered by routines specifically developed by our group as previously described^43,44^.

Mouse Leydig-carcinoma cell line MA-10 (passage number 3 to 8; ATCC®; RRID: CRL-1573) were cultured on 0,1% gelatin/PBS-coated plasticware according to the manufacturer’s instructions and maintained in DMEM:F12 Medium (ATCC®; RRID: 30–2006; pH 7,4) supplemented with additional 20 mM HEPES and horse serum to a final concentration of 15%. For immunofluorescence experiments, cells were let attach on 0,1% gelatin/PBS-coated round-coverslips (ø 13 mm, Menzel-Gläser-VWR International Srl, Milan, Italy) and then washed and fixed with 4% paraformaldehyde/PBS solution at room temperature for 15 min. After membrane permeabilization with 1% Triton X/PBS solution for 10 min at room temperature, cells were saturated with 5% bovine serum albumin/5% normal donkey serum/PBS solution for 1 hour and then incubated with rabbit-polyclonal anti-mouse GPRC6A IgG (dilution 1:200; Santa Cruz Biotechnology Inc. Heidelberg Germany; RRID: sc-67302,) and goat-polyclonal anti-human INSL3 IgG (dilution 1:200; Santa Cruz Biotechnology Inc; RRID: sc-48550) at 4 °C overnight. After extensive washing, primary immunoreaction was detected by appropriate secondary reagent, respectively: biotin conjugated-anti rabbit IgG (dilution 1:100; Vector Laboratories, Peterborough, UK; RRID: BA-1000) followed by Streptavidin TaxasRed, (dilution 1:100; GeneTex/Prodotti Gianni, Milano, Italy; RRID: GTX85907) and FITC conjugated-anti goat IgG (dilution 1:100; Abcam, Milan, Italy; RRID: ab6737). Samples were then counterstained with 4′,6-diamidino-2-phenylindole (DAPI), mounted with anti-fade buffer and then analyzed with Video-Confocal (VICO) fluorescence microscope (Nikon, Firenze, Italy). For stimulation experiments, 60% confluence cells were starved in serum-free medium for 24 hours then exposed for 48 hours to different agonists such as: hCG, ucOCN, SHBG_141–161_ or human SHBG according to experimental conditions detailed below. Testosterone levels in conditioned supernatants were quantified by Testosterone Parameter Assay Kit (R&D System, Abingdon, UK; RRID: KGE010) according to manufacturer’s instructions.

Mouse pancreatic β-cell TC-6 cell line (passage number 19 to 24) was a kind gift of Dr. Stefania Moz (Department of Medicine, University of Padova, Padova, Italy). The cells were maintained in Dulbecco’s Modified Eagle’s medium, (DMEM, ATCC®, cat.# 30-2002) supplemented with 15% fetal bovine serum. For immunofluorescence experiments, cells were let attach on glass round-coverslips coated with 20 mg/mL poly-L-lysine/PBS (Sigma-Aldrich, Milan, Italy). Cells were stained as described above, with the sole exception that primary immune-reaction was performed with rabbit-polyclonal anti-mouse GPRC6A IgG and with rat-anti human/mouse insulin IgG (dilution 1:200; R&D System; RRID: MAB1417) that required appropriate secondary reagent (FITC conjugated-anti mouse IgG; dilution 1:100; Santa Cruz Biotechnology Inc.; RRID: SC-2010). Stimulation experiments were performed as previously described^20,45^. Briefly, 80% confluence cells were starved for 2 hours with HEPES balanced salt solution (HBSS). Cells were then stimulated for 2 hours at 37 °C with aforementioned agonists diluted in 3 mM Glucose/HBSS. Reference stimulating conditions were represented by 15 mM Glucose + 100 μM 3-Isobutyl-1-methylxanthine (IBMX) diluted in HBSS. Secreted insulin was quantified with Insulin Rat/Mouse EIA Kit (DRG Instruments GmbH, Marburg, Germany; RRID: EIA4127) according to manufacturer’s instructions.

Receptor blockade experiments were performed by pre-incubating cultured cells with anti-GPRC6A polyclonal antibodies (final concentration of 20 mg/mL; Santa Cruz Biotechnology Inc; RRID: sc-67302) for 30 minutes at 37 °C as previously described^14^.

Results of binding and stimulation experiments are reported as mean values ± standard deviation (SD) of five independent experiments, expressed as fold increase with respect of basal conditions.

### RNA extraction, cDNA synthesis and real-time PCR

Total RNA was extracted using the RNeasy Mini Kit (Qiagen, Hilden, Germany). cDNA synthesis from total RNA was performed by SuperScript III RT (Invitrogen, Carlsbad, CA, USA) using random hexamers, including a deoxyribonuclease treatment according to manufacturer’s protocol. All isolated RNA was quantified by a Nano-Drop spectrophotometer. Total cDNA was amplified by PCR using following primers for Gprc6a: forward 5′- AGCATTCAGCTTGCAGTGTT -3′and reverse 5′- AAGTAGCTCCCATGGCTGAA -3′. As housekeeping gene, mouse Gapdh was used (forward 5′-GTCGGTGTGAACGGATTTGG-3′ and reverse 5′-CATTCTCGGCCTTGACTGTG-3′). Amplification reactions were performed in a 20 µl final volume containing 20 ng of cDNA, 1X Power SYBR Green PCR Master Mix (Applied Biosystem, Foster City, CA, USA), and a mix of forward and reverse primers (1 mmol/l each). Amplification was carried out with an initial denaturation step at 95° for 10 min followed by 40 cycles of 95° for 15 s, 60° for 30 s, and 72° for 30 s using thermocycler StepOne plus (Applied Biosystems, Foster City, CA, USA). Relative quantification was performed using Delta Delta Ct (ΔΔCt) method. Products of qPCR were checked by gel electrophoresis using 100 bp DNA ladder as marker (Thermo Fisher Scientific Inc, Waltham, MA, USA).

### Statistical analysis

Statistical analysis of the data was conducted with SPSS 21.0 for Windows (SPSS, Chicago, IL). Normal distribution of data was assessed by Kolmogorov-Smirnov test. Two-tailed Student’s-t test was adopted for comparison between two groups, before assessment of normal distribution. Analysis of Variance (ANOVA) with Bonferroni-Holmes correction was adopted for multiple comparisons. Values of P < 0,05 were considered as statistically significant.

## Supplementary information

Supplementary Figures S1 S2 S3
