# Supplementary material for: SHBG141–161 Domain-Peptide Stimulates GPRC6A-Mediated Response in Leydig and β-Langerhans cell lines
Source: Sci Rep. 2019 Dec 19;9:19432. doi: 10.1038/s41598-019-55941-x (PMC6923452; doi:10.1038/s41598-019-55941-x)
Supplement: Supplementary file 1 — Supplementary Figures S1 S2 S3 [file 41598_2019_55941_MOESM1_ESM.pdf]

SHBG141-161 Domain-Peptide Stimulates GPRC6A-Mediated Response in Leydig and β-Langerhans cell lines

Authors: Luca De Toni[a], Diego Guidolin[b], Vincenzo De Filippis[c], Daniele Peterle[c], Maria Santa Rocca[a,d], Andrea Di Nisio[a], Maurizio De Rocco Ponce[a], Carlo Foresta[a]*.

Affiliations

[a] University of Padova, Department of Medicine and Unit of Andrology and Reproductive Medicine, 35128, Padova, Italy

[b] Unversity of Padova, Department of Neuroscience and Section of Anatomy, 35128, Padova, Italy

[c] University of Padova, Department of Pharmaceutical and Pharmacological Sciences, 35131, Padova, Italy.

[d] Familial Cancer Clinic, Veneto Institute of Oncology (IOV-IRCCS), 35128, Padova, Italy

Running Title: Agonist activity of SHBG141-161 on GPRC6A

***Correspondence:**

Prof. Carlo Foresta

Department of Medicine, Unit of Andrology and Reproductive Medicine

University of Padova

Via Giustiniani, 2, 35128 Padova, Italy.

Phone: +39-049-8218517;

Fax: +39-049-8218520;

e-mail: carlo.foresta@unipd.it

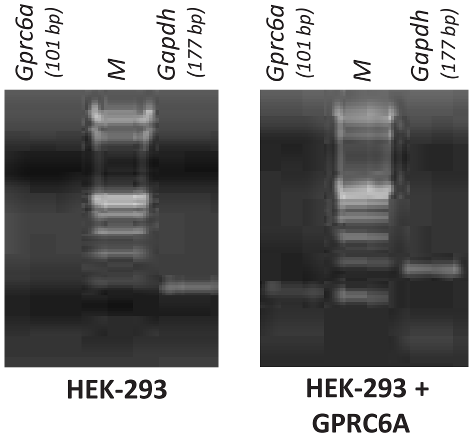

Figure 2 C-I

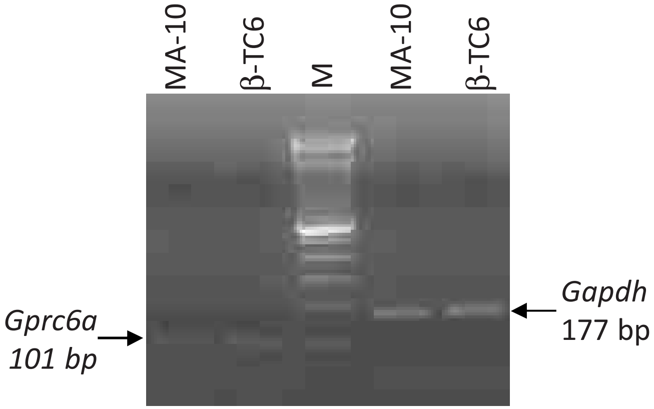

Figure 3 A-I

Supplemental Figure S1

A

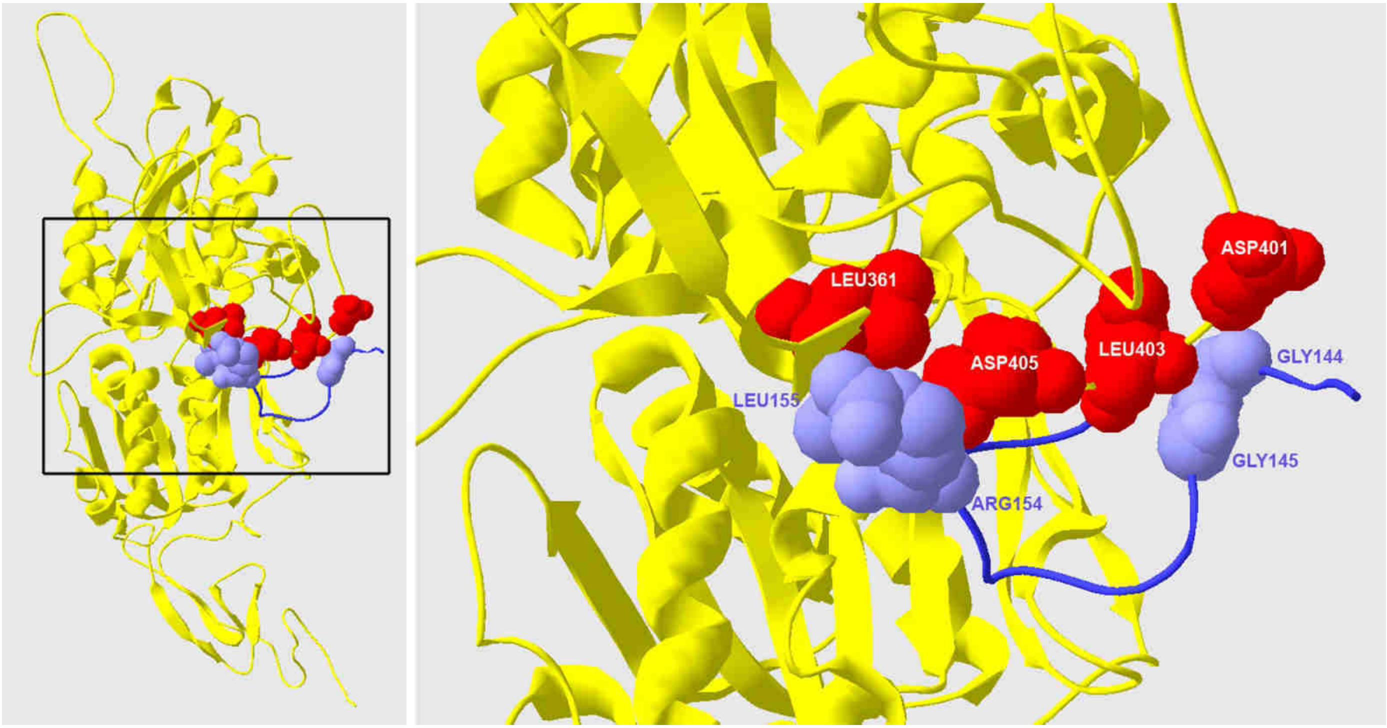

B

GPRC6A	SHGB(141-161)	Distance (Å)
**LEU 403 [N]**	GLY 145 [O]	3.65
**ASP 401 [O]**	GLY 144 [N]	3.53
**ASP 405 [OD2]**	ARG 154 [N]	3.87
**LEU 361 [O]**	ARG 154 [NH1]	3.20
**ASP 405 [OD2]**	LEU 155 [N]	3.18

Supplemental Figure S2

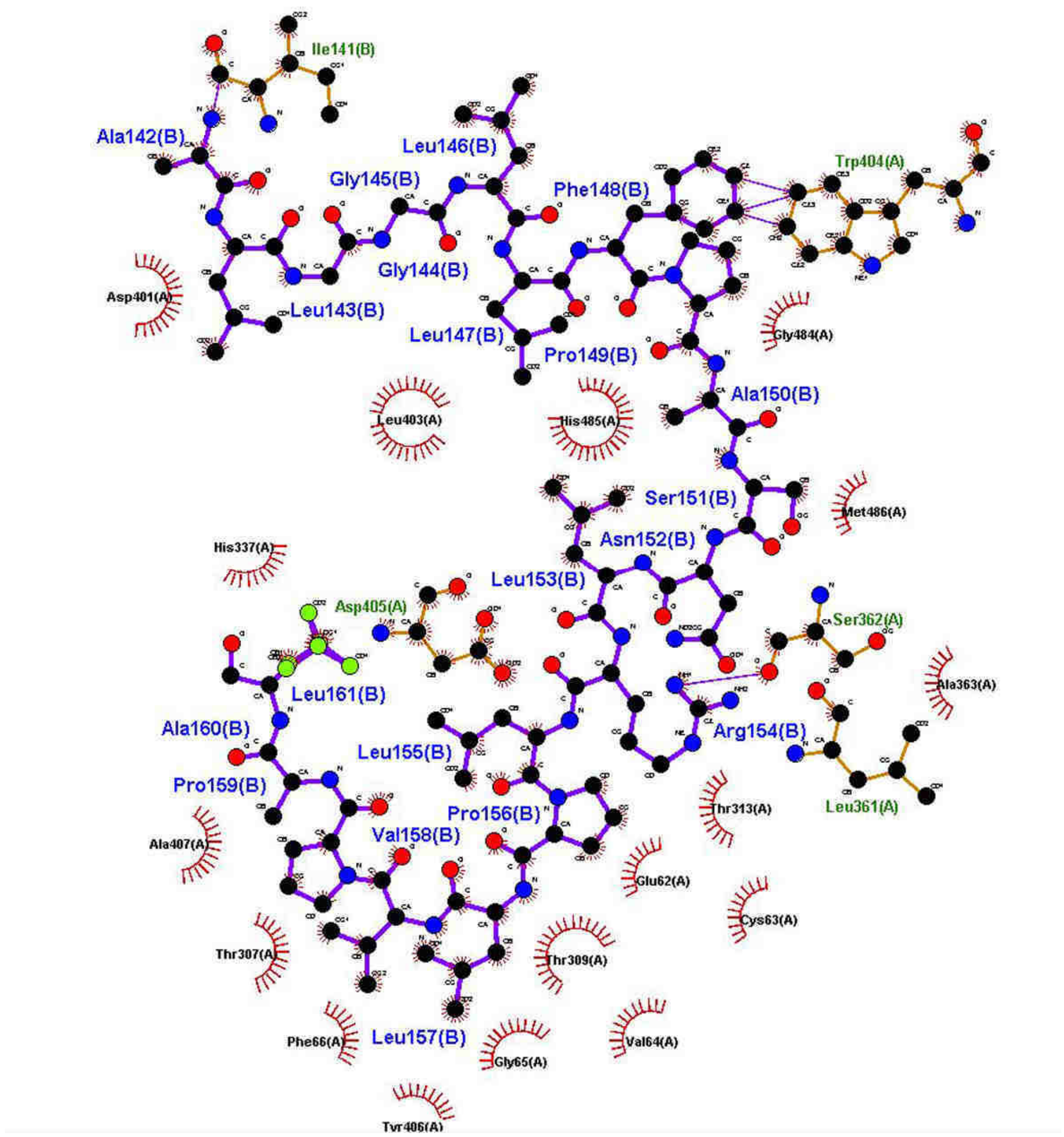

Supplemental Figure S3

Legend to Supplemental Figures

Supplemental Figure S1

Representative images of full-length gels reported in Figure 2 C-I and Figure 3 A-I

Supplemental Figure S2

A) Left panel: Representative image of the extracellular domain of GPRC6A (yellow) bound to $SHBG_{141\text{-}161}$ (blue). In red are represented the residues of GPRC6A predicted to be involved in hydrogen bonds with residues (shown in light blue) of $SHGB_{141\text{-}161}$. Right panel: detail of the interface between GPRC6A and $SHBG_{141\text{-}161}$ where the amino acids predicted to be involved in hydrogen bonds are specified. **(B)** The table provides some more detail on the hydrogen bonds between GPRC6A and $SHBG_{141\text{-}161}$, obtained from PDBePISA server[1].

Supplemental Figure S3

Schematic representation of residues at the interface between GPRC6A and $SHBG_{141\text{-}161}$ where rayed outlines identify amino acids and atoms predicted to be involved in hydrophobic bonds. Chains A and B correspond to GPRC6A and $SHBG_{141\text{-}161}$ respectively. The analysis was performed by using the LigPlot+ software[2].

References

1 Krissinel, E. & Henrick K. Inference of macromolecular assemblies from crystalline state. J Mol Biol. 372(3), 774-97 doi: 10.1016/j.jmb.2007.05.022 (2007)

2 Laskowski, R.A. & Swindells, M.B. LigPlot+: multiple ligand-protein interactions diagrams for drug discovery J Chem Inf Model. 51(10), 2778-86. doi: 10.1021/ci200227u.. (2011)
